# Expression Profile of Human Renal Mesangial Cells Is Altered by Infection with Pathogenic Puumala Orthohantavirus

**DOI:** 10.3390/v14040823

**Published:** 2022-04-15

**Authors:** Christian Nusshag, Lukas Boegelein, Pamela Schreiber, Sandra Essbauer, Anja Osberghaus, Martin Zeier, Ellen Krautkrämer

**Affiliations:** 1 Department of Nephrology, University of Heidelberg, D-69120 Heidelberg, Germany; christian.nusshag@med.uni-heidelberg.de (C.N.); lukasarmin.boegelein@med.uni-heidelberg.de (L.B.); pamela.schreiber@med.uni-heidelberg.de (P.S.); anja.osberghaus@med.uni-heidelberg.de (A.O.); martin.zeier@med.uni-heidelberg.de (M.Z.); 2 Bundeswehr Institute of Microbiology, Department Virology and Intracellular Agents, German Centre for Infection Research, Munich Partner Site, D-80937 Munich, Germany; sandraessbauer@bundeswehr.org

**Keywords:** orthohantavirus, mesangial cell, kidney, glomerulus, proteome, acute kidney injury

## Abstract

Acute kidney injury (AKI) with proteinuria is a hallmark of infections with Eurasian orthohantaviruses. Different kidney cells are identified as target cells of hantaviruses. Mesangial cells may play a central role in the pathogenesis of AKI by regulation of inflammatory mediators and signaling cascades. Therefore, we examined the characteristics of hantavirus infection on human renal mesangial cells (HRMCs). Receptor expression and infection with pathogenic Puumala virus (PUUV) and low-pathogenic Tula virus (TULV) were explored. To analyze changes in protein expression in infected mesangial cells, we performed a proteome profiler assay analyzing 38 markers of kidney damage. We compared the proteome profile of in vitro-infected HRMCs with the profile detected in urine samples of 11 patients with acute hantavirus infection. We observed effective productive infection of HRMCs with pathogenic PUUV, but only poor abortive infection for low-pathogenic TULV. PUUV infection resulted in the deregulation of proteases, adhesion proteins, and cytokines associated with renal damage. The urinary proteome profile of hantavirus patients demonstrated also massive changes, which in part correspond to the alterations observed in the in vitro infection of HRMCs. The direct infection of mesangial cells may induce a local environment of signal mediators that contributes to AKI in hantavirus infection.

## 1. Introduction

Pathogenic Eurasian orthohantaviruses (family *Hantaviridae*) cause hemorrhagic fever with renal syndrome (HFRS) characterized by acute kidney injury (AKI) with often massive proteinuria [1,2]. Disease severity, however, varies between members of the genus orthohantavirus. The latter comprises viruses associated with severe (e.g., Hantaan virus, HTNV; Dobrava-Belgrade virus, DOBV; Sochi virus, SOCV) or mild disease course (PUUV; Kurkino virus, KURV) as well as members that are rarely associated with a mild apparent infection (TULV) or with no known human pathogenic potential [3,4]. In patients with HFRS, hantaviral antigen is detectable in kidney cells and is associated with disruption of tubular and glomerular integrity as shown by light and electron microscopy analyses [2,5,6,7,8,9,10,11]. Direct effects mediated by infection of kidney cells as well as immune-mediated effects may contribute to the clinical picture of hantavirus infection. A plethora of increased cytokines has been described in peripheral blood of hantavirus patients and has been associated with disease severity [12,13,14,15,16,17,18]. In addition, several biomarkers of kidney damage have been identified that may be involved in or predictive for the clinical course. Neutrophil gelatinase-associated lipocalin (NGAL) as a marker of tubular injury is elevated in patients infected with PUUV [19]. Further, the soluble urokinase plasminogen activator receptor (suPAR) and nephrin as markers of glomerular injury are elevated in urine samples of PUUV-infected patients, and levels correlate with the extent of glomerular proteinuria [2,20]. Together, glomerular structural changes and elevation of specific markers such as nephrin and suPAR revealed that glomerular injury contributes to the proteinuria observed in hantavirus infection. However, a detailed analysis of kidney expression profile and the underlying mechanisms contributing to the breakdown of the glomerular filtration barrier in hantavirus infection is lacking.

Proper function of the glomerular units depends on the structural and functional integrity and the accurate interplay between all glomerular cell types [21]. Besides podocytes and glomerular endothelial cells [6,22], mesangial cells may represent relevant hantaviral target cells [23]. Mesangial cells play a major role in inflammatory processes in the kidney by producing cytokines and adhesion molecules [24,25,26]. Transient or productive infection of mesangial cells was already shown for several viruses such as human cytomegalovirus (HCMV), BK virus (BKV), human immunodeficiency virus (HIV), Zika virus, and severe acute respiratory syndrome coronavirus (SARS-CoV) [27,28,29,30,31,32]. Therefore, mesangial infection and changing of the expression profile may play a pivotal role in infectious diseases with kidney involvement.

## 2. Materials and Methods

### 2.1. Cells and Viruses

Primary human renal mesangial cells (HRMC, donor #5948 and #20018) were obtained from Sciencell and maintained in mesangial cell medium (Sciencell, Carlsbad, CA, USA). Human umbilical vein endothelial cells (HUVEC) were obtained from Promocell and maintained in Endothelial Cell Growth Medium (Promocell, Heidelberg, Germany). Conditionally immortalized human podocytes were kindly provided by Jochen Reiser [33] and were maintained in RPMI1640 medium supplemented with 10% FCS and 1% insulin-transferrin-selenium (Invitrogen, Thermo Fisher Scientific, Waltham, MA, USA). Differentiation was induced by shifting to 37 °C. Vero E6 cells (African Green Monkey kidney cells) and CHO cells (Chinese hamster ovary cells) were maintained in DMEM supplemented with 10% FCS. Puumala virus (PUUV) strain Vranica and Tula virus (TULV) strain Moravia were propagated and titrated on Vero E6 cells. Work with orthohantaviruses was carried out in biosafety level 2 containment facilities.

### 2.2. Kinetics of Infection and Release

Cells were inoculated with viruses at a multiplicity of infection (MOI) of 1. After one hour, the viral inoculum was removed by triple washing. Infection kinetics were monitored by immunofluorescence staining of nucleocapsid protein (N protein) and quantification of the percentage of positive cells. To quantify the release of infectious particles, single-round infection assay in Vero E6 cells was performed [34]. In brief, cell-free supernatants from infected human mesangial cells were collected on day two, four, and six after infection, and cell-free supernatants were added to Vero E6 cells for two hours. Inoculum was removed by triple washing with medium. After 48 h and before viral spread started, the total number of initially infected Vero E6 cells was quantified by immunofluorescence staining of N protein. Titers were expressed as infectious units (IU) per ml supernatant. All experiments were performed in triplicates.

### 2.3. Immunofluorescence and Western Blot

Cells grown on coverslips were fixed with 3% paraformaldehyde and stained with primary and fluorescently labeled secondary antibodies. The following antibodies were used: mouse anti-α-smooth muscle actin (α-SMA) (clone 1A4, Sigma, Darmstadt, Germany), mouse anti-synaptopodin (clone D-9, Santa Cruz, Santa Cruz, CA, USA), mouse anti-cytokeratin 18 (CK18) (clone RGE-53, Merck Millipore, Darmstadt, Germany), rabbit anti-fibronectin (Sigma), mouse anti-integrin α_v_β_3_ (clone LM609, Millipore), mouse anti-CD31 (Dako, Glostrup, Denmark), and mouse anti-N protein PUUV (A1C5, Progen, Heidelberg, Germany). Cell nuclei were stained by Hoechst 33342 (Invitrogen, Thermo Fisher Scientific, Waltham, MA, USA). Images were taken using an Axiocam 506 mono camera attached to an Axio Observer.D1 inverted microscope (Carl Zeiss, Oberkochen, Germany). For Western blot analysis, the following primary antibodies were used: rabbit anti-PUUV N protein and mouse anti-α-tubulin (Sigma). Loading was verified by the detection of tubulin on the same membrane. Detection was performed by using near infrared fluorescent dye (IRDye)-conjugated secondary antibodies and an Odyssey CLx infrared imaging system (Li-Cor, Lincoln, NE, USA).

### 2.4. Flow Cytometry Analysis

Cells were washed with phosphate buffered saline (PBS), scraped and stained with allophycocyanin (APC)-conjugated anti-CD55 (clone IA10, BD Pharmingen, Heidelberg, Germany) and phycoerythrin (PE)-conjugated mouse anti-integrin α_V_β_3_ antibody (clone LM609, Millipore). For detection of integrin β_1_, cells were stained with PE-conjugated mouse anti-integrin β_1_ antibody (clone P5D2, R&D Systems, Wiesbaden, Germany). Corresponding isotype controls served as controls. After one hour of incubation on ice, the cells were washed and analyzed by flow cytometry with FACSCalibur (BD Pharmingen). Flow cytometry analysis of cell viability was monitored by using Via-Probe™ Cell Viability Solution (BD Pharmingen) according to manufacturer’s instructions.

### 2.5. Viability Assay

Uninfected and infected cells were lysed 4 days post-infection (dpi). The number of viable cells was determined by measuring the amount of ATP using CellTiter-Glo luminescent cell viability assay (Promega, Walldorf, Germany).

### 2.6. Motility Assay

Uninfected and infected HRMCs (10,000 cells/cm^2^) on μ-slide 8-wells (Ibidi) were subjected to live cell imaging on 4 dpi. The motility of cells was monitored for six hours by a JuLi Smart Fluorescence Cell Imager (NanoEnTek, Waltham, MA, USA). In each experiment, 30 cells were tracked by the ImageJ manual tracking plugin (Ibidi, Gräfelfing, Germany), and statistical analysis was done by using the chemotaxis tool plugin (Ibidi).

### 2.7. Adhesion Assay

Single-cell suspensions of 10,000 uninfected or infected HRMCs (4 dpi) were added in each well of a 96-well microtiter plate and left to adhere for 30 min at 37 °C. After a triple washing with PBS, adhered cells were fixed, stained with Sapphire700 (Li-Cor) and DRAQ5 (BioStatus), and quantified via scanning with an Odyssey CLx infrared imaging system (Li-Cor).

### 2.8. Patient Samples

Patients with serologically confirmed (*recom*Line HantaPlus assay, Mikrogen Diagnostik) acute PUUV infection (*n* = 11) hospitalized in the Department of Nephrology, University of Heidelberg, Germany were included. There were no records of any previous kidney diseases. Patient characteristics and clinical data were obtained from medical charts. For a control, urine samples of seven healthy-age and gender-matched persons were analyzed. Written informed consent was obtained from all participants. This study was approved by the Ethics Committee of the University of Heidelberg, Heidelberg, Germany, and it adhered to the Declaration of Helsinki.

### 2.9. Proteome Profiler

Proteome profiles of urinary samples derived from PUUV-infected and uninfected healthy individuals or cell-free supernatants derived from uninfected and PUUV-infected HRMCs (4 dpi) were analyzed by a Human Kidney Biomarker Array Kit (R&D Systems) according to manufacturer’s instructions. In brief, urine or cell culture supernatant samples together with biotinylated detection antibodies were added to membranes spotted with antibodies against 38 different kidney protein markers in duplicates. Detection of spot signal intensities, corresponding to the amount of bound proteins, was performed by using near infrared fluorescent IRDye800CW streptavidin and by scanning with an Odyssey CLx infrared imaging system (Li-Cor).

### 2.10. Statistical Analysis

Statistical analysis was performed using Prism 5.0 (Graphpad Software Inc., San Diego, CA, USA). Normal distribution was tested by the Kolmogorov–Smirnov test. Values of two groups were compared using two-tailed Student’s *t*-test or the Mann–Whitney test. *p* values of <0.05 were considered significant. ns: not significant; * *p* < 0.05; ** *p* < 0.01; *** *p* < 0.005; **** *p* < 0.0001.

## 3. Results

### 3.1. Characterization of Cells

**Figure 1:**
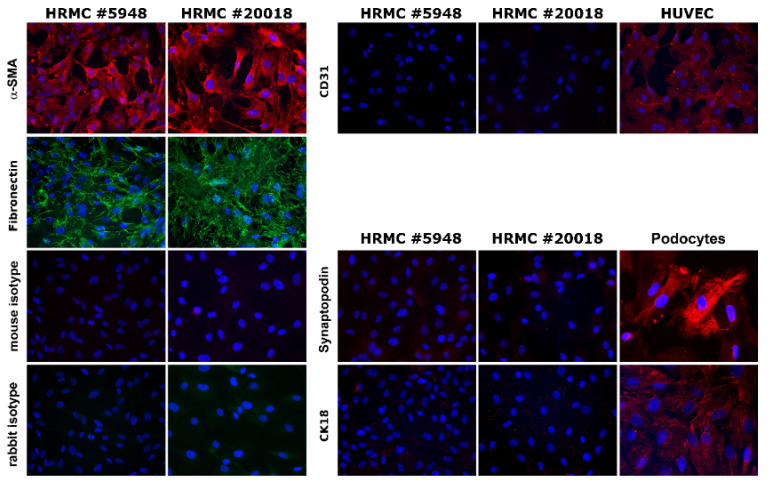
Characterization of HRMCs of two donors. HRMCs were characterized by immunofluorescence for cell-type specific marker proteins. HUVECs served as positive control for endothelial marker protein CD31, and podocytes were identified by staining with epithelial marker CK18 and podocyte-specific protein synaptopodin. Rabbit and mouse isotype control were tested on the same slide for donor#5948 using the corresponding near infrared fluorescent dye (IRDye)-conjugated secondary antibodies.

First, we examined HRMCs for cell-type-specific marker proteins and receptor expression. We analyzed cells from two different donors. All cells exhibited the expression pattern specific for renal mesangial cells: Expression of the podocyte-specific protein synaptopodin, the endothelial marker CD31, and the epithelial marker cytokeratin 18 was completely absent, whereas expression of α-SMA and fibronectin that are typically expressed in mesangial cells, was detectable in the isolated HRMCs [35] (Figure 1).

### 3.2. Infection of HRMCs with PUUV

**Figure 2:**
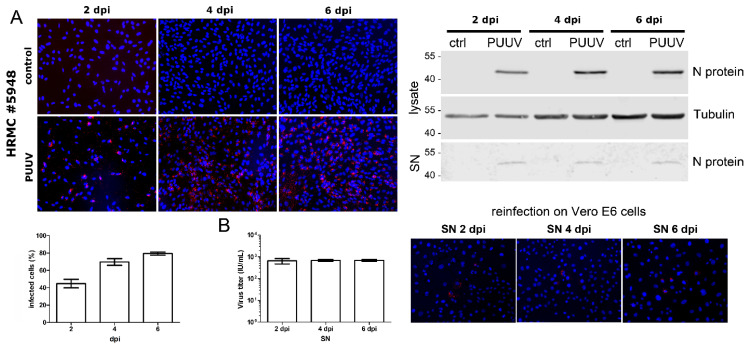
Infection of HRMCs with PUUV. HRMCs were infected with PUUV at an MOI of 1. Infection was monitored by detection of N protein by immunofluorescence and by Western blot analysis of lysates and cell-free supernatants (SN) at the indicated time points post-infection (**A**). Titers of infectious units in the supernatant were determined by a single-round infection assay in Vero E6 cells (**B**). Three independent experiments were performed. The mean ± standard deviation (SD) is shown.

We examined the permissivity of HRMCs for PUUV (Figure 2). Cells were inoculated with PUUV, and infection was monitored by detection of N protein via immunofluorescence and Western blot. The percentage of infected cells was increasing over time. At day six post-infection, 79.43% of the cells were infected. Release of particles was analyzed by detection of N protein in cell-free supernatants and inoculation of Vero E6 cells with cell-free supernatants derived from infected HRMCs. Levels of 704 IU/mL were detected in the supernatant on 6 dpi. Viral spread in HRMCs and the detection of infectious particles in the supernatant demonstrate the productive infection of mesangial cells by PUUV.

### 3.3. Infection of HRMCS with TULV

**Figure 3:**
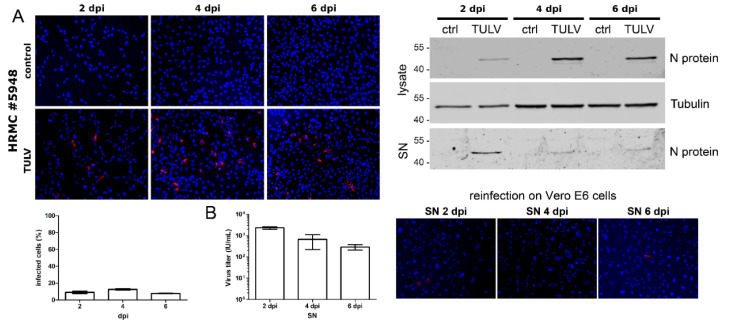
Infection of HRMCs with TULV. HRMCs were inoculated with TULV at an MOI of 1 and analyzed for N protein expression by immunofluorescence and by Western blot analysis of lysates and SN at the indicated time points (**A**). Release of infectious units was quantified by a single round infection assay on Vero E6 cells (**B**). Three independent experiments were performed. The mean ± SD is shown.

In a next step, we analyzed the ability of low-pathogenic TULV to replicate in HRMCs. An initial infection of about 10% of cells was observed on day two post-infection. Despite detection of the TULV N protein and particles infecting Vero E6 in the supernatant, there was only a very small increase in the number of infected HRMCs between day two and four. In contrast to of PUUV, the infection of HRMCs with TULV was abortive, and no extensive viral spread was observable (Figure 3).

**Figure 4:**
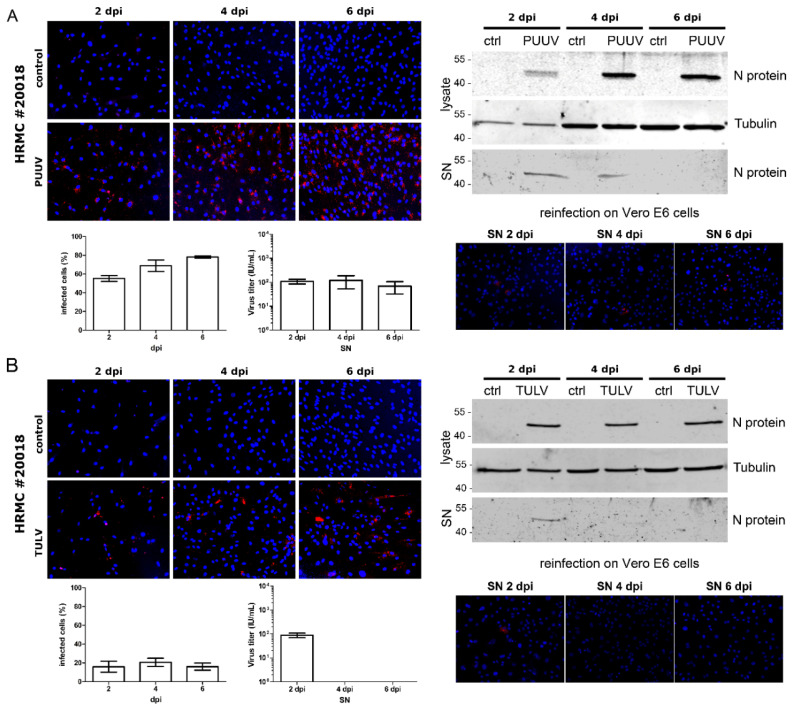
Infection of HRMCs derived from a second donor with PUUV and TULV. HRMCs were inoculated with PUUV (**A**) or TULV (**B**) at an MOI of 1 and analyzed for N protein expression by immunofluorescence and by Western blot analysis of lysates and SN at the indicated time points. Release of infectious units was quantified by a single round infection assay on Vero E6 cells. Three independent experiments were performed for each virus. The mean ± SD is shown.

To exclude possible donor-specific effects, we tested the infection with PUUV and TULV in HRMCs of a second donor (Figure 4). As observed for the first donor, PUUV replicated very efficiently in HRMCs, and infectious particles were released (Figure 4A). In contrast, only a small amount of cells was infected with TULV at day two with a slight increase on 4 dpi (Figure 4B). Release of infectious particles was detected only on day two post-infection. Infection experiments of HRMCs of two donors with TULV indicated that only a small fraction of HRMC population was permissive for TULV infection and that the viral spread in HRMCs was negligible or absent despite the detection of infectious particles in the reinfection of Vero E6 cells with supernatants derived from infected HRMCs.

### 3.4. Expression of Integrin β_3_ and β_1_ as Hantaviral Receptors

**Figure 5:**
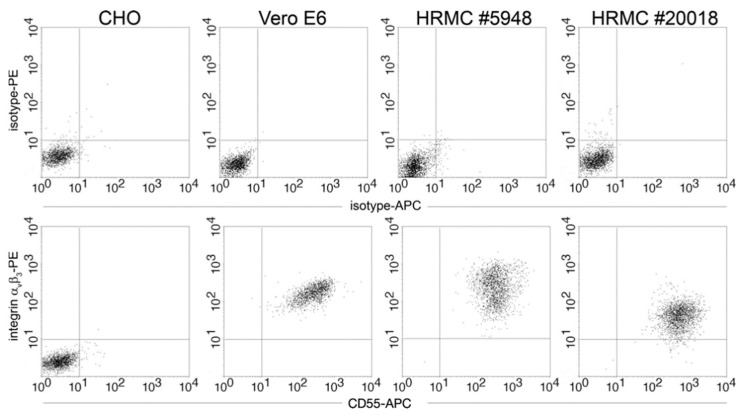
Receptor expression in primary human mesangial cells. Surface expression of hantaviral receptor proteins integrin α_v_β_3_ and CD55 was analyzed in two donors by flow cytometry. CHO cells served as negative control and Vero E6 cells as positive control for integrin α_v_β_3_ expression. Upper panels: isotype controls, lower panels: receptor staining.

Surface expression of integrin α_v_β_3_ and CD55, which were described to be involved in the entry of pathogenic hantaviruses [36,37], was analyzed by flow cytometry. HRMCs of both donors showed surface expression of both proteins (Figure 5).

**Figure 6:**
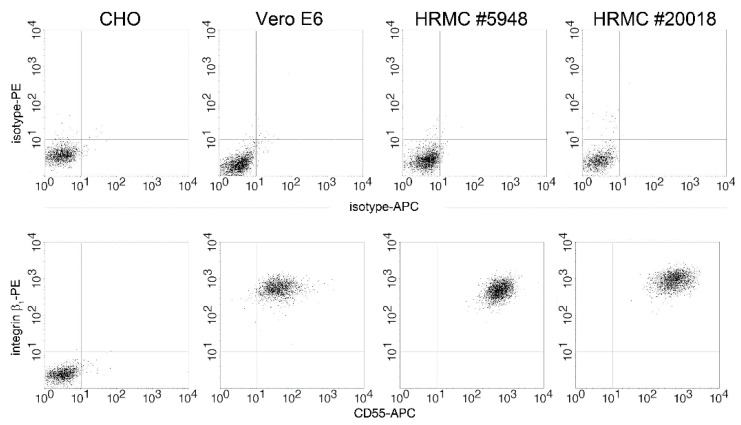
Detection of integrin β_1_ expression in HRMCs of two donors. Surface expression of receptor integrin β_1_ and CD55 was analyzed by flow cytometry. Upper panel: isotype control, lower panel: receptor staining. CHO and Vero E6 cells served as negative and positive control for integrin β_1_ expression, respectively.

TULV was defined as non- or low-pathogenic virus and is supposed to enter cells via the integrin β_1_ receptor [38,39]. Therefore, we analyzed the surface expression of integrin β_1_ on HRMCs (Figure 6). Expression of integrin β_1_ on the total HRMC population of both donors was observed.

### 3.5. Functional Consequences in PUUV-Infected HRMCs

**Figure 7:**
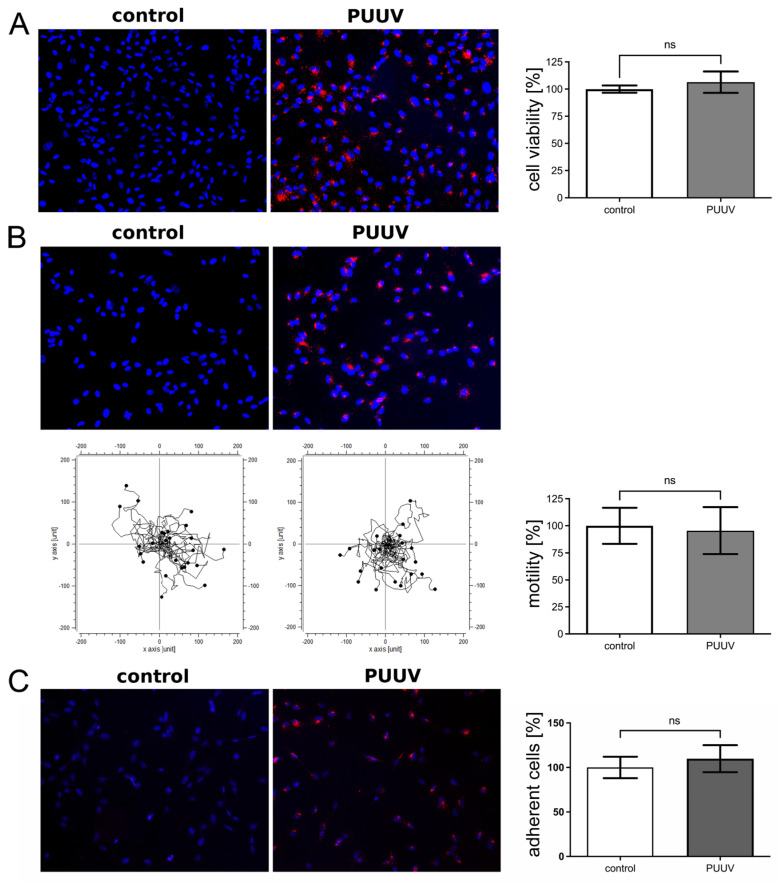
Analysis of functional consequences in PUUV-infected mesangial cells. Viability (**A**), migration capacity (**B**) and adhesion (**C**) of PUUV-infected HRMCs (4 dpi) were analyzed. Infection was quantified by staining of N protein. Results of uninfected cells were set to 100%. Three independent experiments were performed. The mean ± SD is shown. ns: not significant.

Infection may influence the viability of cells. Viability of infected glomerular endothelial cells, podocytes, and tubular epithelial cells was not impaired, but effects on adhesion and migration capacity were described [40,41]. Therefore, we examined PUUV-infected HRMCs for impairment of cellular functions (Figure 7). For functional assays, more than 98% of HRMCs were infected as demonstrated by detection of N protein expression by immunofluorescence (Figure 7A–C). Neither viability nor capacity of migration and adhesion were significantly impaired by infection with PUUV. Due to the very low number of cells infected with TULV even at higher MOIs, the analysis of functional effects was not possible.

### 3.6. Analysis of Proteome Profile in Supernatants of Infected HRMCs

**Figure 8:**
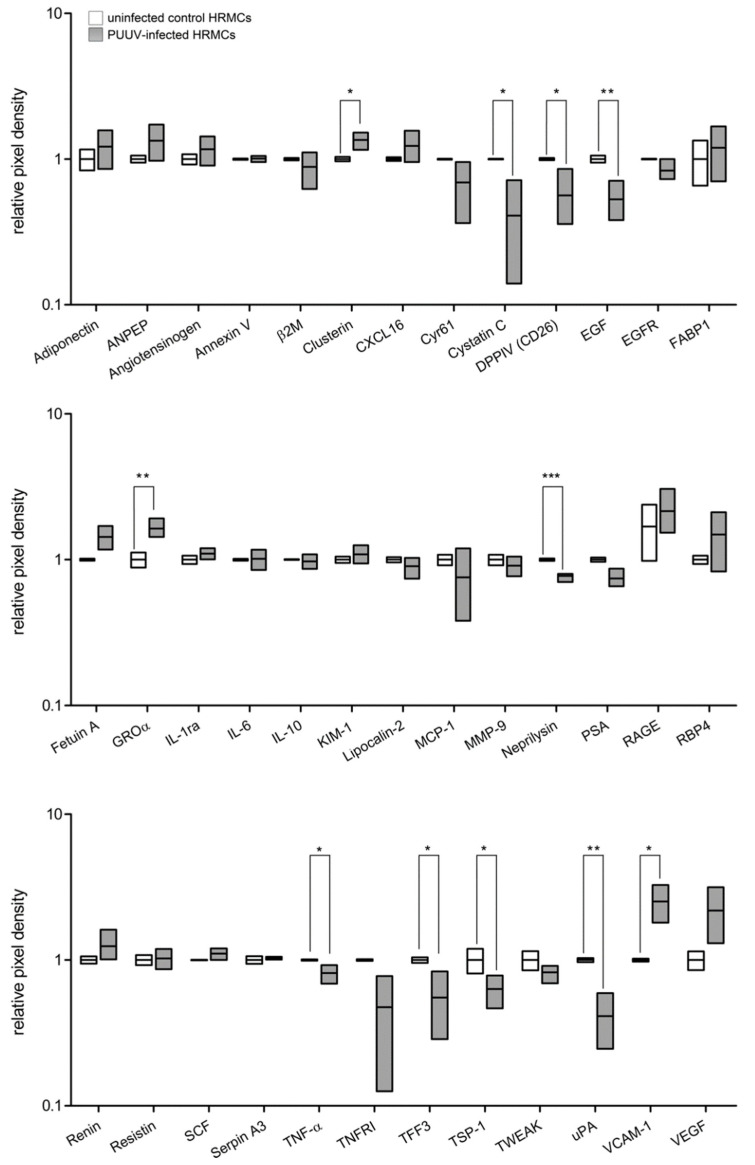
Proteome profile of PUUV-infected HRMCs. Cell-free cell culture supernatant was collected from mock-infected cells and cells infected with PUUV at day 4 post-infection. Levels of relative pixel intensity of uninfected HRMCs were set to 1. The minimal and maximal levels are shown, the lines indicate the mean level. Two independent experiments were performed in duplicates. * *p* < 0.05; ** *p* < 0.01; *** *p* < 0.005.

Mesangial cells play a central role in inflammatory processes and in the communication between cell types within the glomerulus. Therefore, we analyzed the proteome profile of PUUV-infected HRMCs (Figure 8). The analysis demonstrated changes of kidney biomarkers. In supernatants of infected cells, 11 of 38 analyzed proteins revealed altered levels. Levels of eight proteins were reduced, and three proteins exhibited increased levels compared to those of uninfected cells. The deregulation involves angiogenic, inflammatory and adhesion factors, and proteases. Upregulation of clusterin, growth-regulated oncogene α (Groα), and vascular cell adhesion molecule-1 (VCAM-1) and downregulation of cystatin C, dipeptidyl peptidase IV (DPPIV), epidermal growth factor (EGF), neprilysin, tumor necrosis factor-α (TNF-α), trefoil factor 3 (TFF3), thrombospondin-1, and urokinase-type plasminogen activator (uPA) were observed.

### 3.7. Urinary Proteome Profile of Patients with Acute Hantavirus Infection

**Figure 9:**
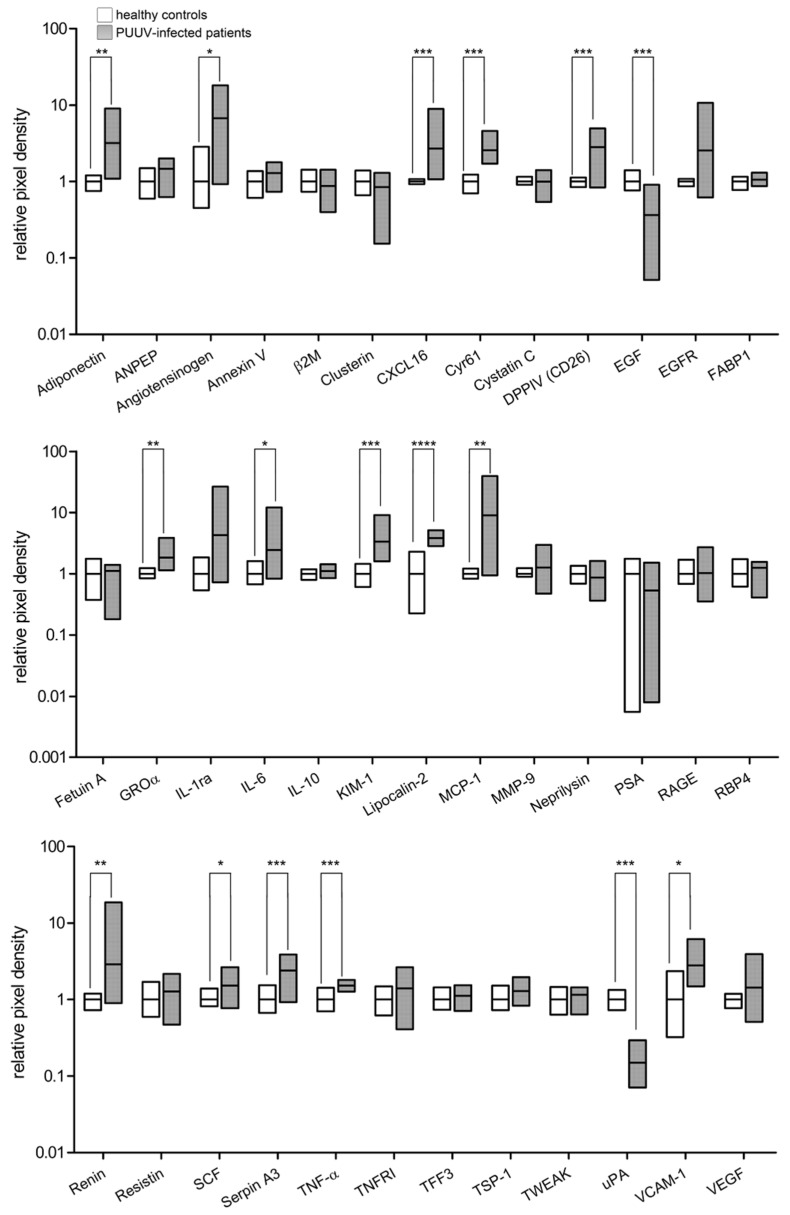
Urinary proteome profile of patients with acute PUUV hantavirus infection. Urine samples of eleven patients and an age- and gender-matched healthy control group (*n* = 7) were analyzed by a proteome profiler. Bars show minimal and maximal values of relative pixel intensity. Lines indicate mean relative pixel intensity. Mean of controls was set to 1. * *p* < 0.05; ** *p* < 0.01; *** *p* < 0.005; **** *p* < 0.0001.

To characterize the role of mesangial cells in the overall changes of kidney proteome profile in infected patients, we also analyzed the urinary proteome profile of eleven patients with acute hantavirus infection by proteome profiler assay. Urinary samples were collected at day 9.6 ± 3.5 (mean ± SD) after onset of symptoms (Figure 9). All patients showed characteristic alterations in laboratory parameters of acute hantavirus infection: elevation of leukocyte count, serum creatinine, C-reactive protein (CRP) level and lactate dehydrogenase (LDH) activity, as well as a decrease in platelet counts (Table 1). A semiquantitative analysis via urine dipstick revealed proteinuria in all patients (range 0.3–> 10.0 g/L).

Levels of urinary markers were compared with an age- and gender-matched healthy control group. In PUUV-infected patients, the relative abundance of 17 of 38 analyzed markers was changed compared to that in healthy controls. We observed an increase for 15 markers and a decrease for two marker proteins in the urine samples. Different cytokines, chemokines, and marker of renal cell damage were elevated, and levels of uPA and EGF were reduced. Levels of six proteins altered in patients were also affected in in vitro-infected HRMCs. Both profiles were characterized by a decrease of EGF and uPA and upregulation of Groα and VCAM-1. In contrast, DPPIV and TNF-α were increased in patients and decreased in in vitro HRMC infection. Levels of other markers exhibited similar trends in patients and cell culture infection, however, without reaching statistical significance.

## 4. Discussion

Kidney involvement in infectious diseases is frequently observed, and the pathogenesis is difficult to elucidate due to the multiple effects that may contribute to kidney injury. Target cells, direct effects, and release of soluble mediators determine the clinical picture of an infectious disease. For the Eurasian hantavirus infection, kidney manifestations are characteristic and may be caused by direct infection of kidney cell types and the establishment of an inflammatory environment.

We identified a significant change in the proteome profile of urine samples of patients with acute hantavirus infection compared to that of healthy persons. Markers of tubular damage such as adiponectin, angiotensinogen, kidney injury molecule-1 (KIM-1), and lipocalin-2 were elevated. In line with our data, Bunz et al. recently described urinary lipocalin-2 (NGAL) as a predictor of disease severity in hantavirus infection [19]. Furthermore, levels of proinflammatory cytokines, chemokines, proteases, and marker of cellular damage were increased. The upregulation of some of these proteins such as IL-6, TNF-α, and VCAM-1 have been demonstrated in kidney biopsy and urine samples of HFRS-patients and were identified to correlate with disease severity and proteinuria [42,43]. Therefore, the infection of kidney cells by pathogenic hantaviruses and the specific induction of a kidney inflammasome may contribute to the characteristic renal syndrome in hantavirus disease.

Our study demonstrates the productive infection of mesangial cells by pathogenic PUUV. In contrast, non- or low-pathogenic TULV is restricted to an initial and abortive infection of a minor percentage of the mesangial cell population. These results are in accordance with the absence of TULV replication in human PMA-differentiated monocytes (THP-1/PMA), microvascular endothelial cells (HMEC-1), and glomerular endothelial cells [22]. The mechanisms of TULV restriction are not clear. As observed for THP-1/PMA cells and HMEC-1 by Bourquain et al., HRMCs express integrin β_1_, but TULV replication is inefficient or absent. However, entry and receptor usage of orthohantaviruses is not completely understood, and an increasing number of proteins is described to mediate hantaviral entry [37,44,45,46,47,48,49]. Future investigations are necessary to identify relevant factors of susceptibility and permissivity for pathogenic and nonpathogenic members in kidney target cells, because direct infection of different cell types with pathogenic hantaviruses may play a role in the characteristic kidney-specific clinical picture of HFRS.

Interestingly, PUUV infection of HRMCs does not result in a decrease of migration and adhesion capacity as it was observed for podocytes, glomerular endothelial, and tubular epithelial cells, indicating cell-type specific differences in infection of renal cells [40]. However, the infection with PUUV led to significant changes in the proteome profile of mesangial cells. Levels of proteases, angiogenic, adhesion, and proinflammatory factors were altered compared to those in uninfected cells. Levels of six proteins that were changed in infected mesangial cells were also affected in urine samples of patients with acute PUUV infection. We identified similarities and possible key players in the proteome profiles: levels of Groα and VCAM-1 were elevated, EGF and uPA were decreased. These markers were described to play a role in AKI pathogenesis of various origins. The up-regulation of mesangial Groα and VCAM-1 expression contributes to immune cell infiltration of the glomeruli [26,50]. Upregulation of Groα transcription is also observed in HTNV-infected HUVECs [51]. The transient downregulation of urinary EGF is observed in patients with AKI in models of glomerular and tubular injury and is proposed to be a prognostic marker of AKI [52,53]. The role of decreased uPA levels is of special interest. Levels of PAI-1 and uPA in HCPS were previously analyzed by Simons et al., who observed that plasma levels of total uPA are lower in survivors [54]. However, data on uPA in HFRS patients are lacking. In vitro studies demonstrate that uPA is not downregulated in PUUV-infected blood microvascular endothelial cells [55]. Therefore, the cell type-specific downregulation of uPA in mesangial cells may be of special pathophysiological interest.

However, there are also differences between the profiles of patients and in vitro-infected HRMCs indicating that the involvement of other renal cell types and the immune system plays a crucial role in kidney pathogenesis. Most altered marker protein levels were decreased in in vitro-infected HRMCs compared to those in uninfected cells, whereas in patients, the majority of proteins were elevated. Possibly, the time point of analysis may contribute to the differences, and additional kinetic studies in patient samples and in in vitro-infected HRMCs from different donors are required to gain insights in the spatiotemporal kidney environment.

It is known that the integrity of the glomerular filtration barrier depends on the complex interaction between kidney cell types. Further investigations are needed to analyze the complex local kidney inflammasome and to elucidate the contribution and interplay of different kidney cell types to hantavirus pathogenesis. Besides the cell-type specific effects, the virus-specific characteristics of hantavirus infection are of particular interest. Hantaviruses exhibit a broad range of pathogenicity despite their high genetic similarity. In vitro studies in HUVECs and the pulmonary cell line A549 identified specific transcription profiles for infections with hantaviruses corresponding to their virulence [51,56]. Therefore, it would be of interest to analyze the consequences of infection on the expression profile in more kidney cell types and with hantaviruses of different pathogenicity.

## 5. Conclusions

Acute hantavirus infection results in massive changes of the urinary proteome in vivo. In vitro, mesangial cells are permissive to PUUV, and infection results in overlapping similarities in the proteome profile compared to that of human urine samples. Therefore, direct infection of kidney cells by pathogenic hantaviruses may contribute to the acute kidney injury in hantavirus disease. Further examination of kidney cell type-specific permissivity and elucidation of signal cascades in infected cells will provide useful insights into the complex kidney pathogenesis of hantavirus infection.

## Figures and Tables

**Table 1:** Characteristics, mean peak and nadir levels of laboratory parameters of eleven patients with serologically confirmed acute PUUV infection.

Characteristic	Mean Value ± SD	Reference Value
Age (years)	40.55 ± 15.36	-
Duration of hospitalization (days)	6.55 ± 2.81	-
Max serum creatinine (mg/dL)	6.74 ± 2.37	0.6–1.2
Min serum albumin level (g/L)	31.72 ± 1.67	30–50
Min platelet count (×10^9^/L)	92.64 ± 45.89	150–440
Max leukocyte count (×10^9^/L)	11.93 ± 2.38	4–10
Max CRP level (mg/L)	68.09 ± 32.02	<5
Max LDH activity (U/L)	431.8 ± 70.96	<317

## Data Availability

Not applicable.

## Abbreviations

α-SMA	α-smooth muscle actin
AKI	acute kidney injury
ANPEP	aminopeptidase N
APC	allophycocyanin
β2M	β2-microglobulin
BKV	BK virus
CHO	Chinese hamster ovary
CK18	cytokeratin 18
CRP	C-reactive protein
CXCL16	CXC-motif ligand 16
Cyr61	cysteine-rich angiogenic inducer 61
DOBV	Dobrava-Belgrade virus
dpi	days post infection
DPPIV	dipeptidyl peptidase IV
EGF	epidermal growth factor
EGFR	EGF receptor
FABP1	fatty acid binding protein 1
Groα	growth-regulated oncogene α
HCMV	human cytomegalovirus
HCPS	hantaviral cardiopulmonary syndrome
HFRS	hemorrhagic fever with renal syndrome
HIV	human immunodeficiency virus
HMEC-1	human microvascular endothelial cell-1
HRMC	human renal mesangial cell
HTNV	Hantaan virus
HUVEC	human umbilical vein endothelial cell
IL	interleukin
IL-1ra	IL-1 receptor antagonist
IU	infectious units
KIM-1	kidney injury molecule-1
KURV	Kurkino virus
LDH	lactate dehydrogenase
MCP-1	monocytes chemoattractant protein-1
MMP-9	matrix metallopeptidase-9
MOI	multiplicity of infection
N protein	nucleocapsid protein
NGAL	neutrophil gelatinase-associated lipocalin
ns	not significant
PAI-1	plasminogen activator inhibitor-1
PBS	phosphate buffered saline
PE	phycoerythrin
PMA	phorbol myristate acetate
PSA	prostate specific antigen
PUUV	Puumala virus
RAGE	receptor for advanced glycation end products
RBP4	retinol binding protein 4
SARS-CoV	severe acute respiratory syndrome coronavirus
SCF	stem cell factor
SD	standard deviation
SOCV	Sochi virus
suPAR	soluble urokinase plasminogen activator receptor
TFF3	trefoil factor 3
TNF-α	tumor necrosis factor-α
TNFR1	TNF receptor type 1
TSP-1	thrombospondin-1
TULV	Tula virus
uPA	urokinase-type plasminogen activator
TWEAK	tumor necrosis factor-like weak inducer of apoptosis
VCAM-1	vascular cell adhesion molecule-1
VEGF	vascular endothelial growth factor
